# *k*-mer-Based Metagenomics Tools Provide a Fast and Sensitive Approach for the Detection of Viral Contaminants in Biopharmaceutical and Vaccine Manufacturing Applications Using Next-Generation Sequencing

**DOI:** 10.1128/mSphere.01336-20

**Published:** 2021-04-21

**Authors:** Madolyn L. MacDonald, Shawn W. Polson, Kelvin H. Lee

**Affiliations:** a Department of Chemical and Biomolecular Engineering, University of Delaware, Newark, Delaware, USA; b Ammon Pinizzotto Biopharmaceutical Innovation Center, Newark, Delaware, USA; c Center for Bioinformatics and Computational Biology, University of Delaware, Newark, Delaware, USA; d Department of Computer and Information Sciences, University of Delaware, Newark, Delaware, USA; Georgetown University

**Keywords:** next-generation sequencing, viral metagenomics, Chinese hamster ovary cells, HeLa cells, adventitious agent testing, vaccine, virus detection

## Abstract

Adventitious agent detection during the production of vaccines and biotechnology-based medicines is of critical importance to ensure the final product is free from any possible viral contamination. Increasing the speed and accuracy of viral detection is beneficial as a means to accelerate development timelines and to ensure patient safety. Here, several rapid viral metagenomics approaches were tested on simulated next-generation sequencing (NGS) data sets and existing data sets from virus spike-in studies done in CHO-K1 and HeLa cell lines. It was observed that these rapid methods had comparable sensitivity to full-read alignment methods used for NGS viral detection for these data sets, but their specificity could be improved. A method that first filters host reads using KrakenUniq and then selects the virus classification tool based on the number of remaining reads is suggested as the preferred approach among those tested to detect nonlatent and nonendogenous viruses. Such an approach shows reasonable sensitivity and specificity for the data sets examined and requires less time and memory as full-read alignment methods.

**IMPORTANCE** Next-generation sequencing (NGS) has been proposed as a complementary method to detect adventitious viruses in the production of biotherapeutics and vaccines to current *in vivo* and *in vitro* methods. Before NGS can be established in industry as a main viral detection technology, further investigation into the various aspects of bioinformatics analyses required to identify and classify viral NGS reads is needed. In this study, the ability of rapid metagenomics tools to detect viruses in biopharmaceutical relevant samples is tested and compared to recommend an efficient approach. The results showed that KrakenUniq can quickly and accurately filter host sequences and classify viral reads and had comparable sensitivity and specificity to slower full read alignment approaches, such as BLASTn, for the data sets examined.

## INTRODUCTION

While virus identification and clearance are required during the manufacturing of vaccines, biologics, and biotechnology-based medicines, several virus contamination events have occurred in both vaccine and biotherapeutic protein production. Since 1988, at least 14 contamination events in Chinese hamster ovary (CHO) cells, the preferred platform for monoclonal antibody production, have been reported (1). These included contamination with minute virus of mice (MVM) at several different companies (2, 3), including an event at Genentech in 2010 that cost millions of dollars (www.sigmaaldrich.com/technical-documents/articles/biology/viral-invaders.html). Other contamination events involved Cache Valley virus (4), vesivirus 2117 (5), reovirus (4), and epizootic hemorrhagic disease virus (6). The predicted sources of contamination in these cases were raw materials (4, 7, 8). Four major cases of viral contamination in vaccines have been reported since 1960 (9), including simian virus 40 found in polio vaccines in 1960, bacteriophages found in measles and polio vaccines in 1973, and porcine circovirus-1 (PCV1) DNA which was found, using sequencing and microarrays, in a rotavirus vaccine in 2010 due to PCV1 contamination (10). In addition, a novel rhabdovirus has been previously identified in Sf9 cell lines, which are used for the production of two vaccines in the United States (11). Although these contamination events are rare, they can have significant economic consequences when they do occur and highlight the need for more frequent and more accurate viral testing during the production of biologics.

Currently, different stages of the manufacturing process are tested for viral contaminants using either *in vivo* or *in vitro* assays (12–14). While beneficial and well established, these traditional viral detection methods have several disadvantages. First, most methods are time-consuming, taking 14 to 28 days to complete (13, 15, 16). During *in vitro* testing, bulk harvest material is inoculated onto detector cells that are then observed after 2 to 4 weeks for signs of viral infection (13, 15). *In vivo* tests are also lengthy processes where the sample of interest is inoculated into virus-free animals, and the animals are then observed over several weeks. There are also *in vivo* species-specific antibody production tests where the serum antibody levels are measured after a specified period of time (12, 13, 17). Second, an alternate method, PCR-based identification, requires the design of primers for a specific type of virus before it can be identified, and therefore, this method requires prior knowledge of possible contaminants (1, 18). To overcome these challenges, next-generation sequencing (NGS) methods have recently been proposed as an alternative virus detection strategy (19–23). NGS can be much faster, with the sequencing only taking 1 to 2 days, although the sample processing and bioinformatics analysis can take anywhere from a few hours to weeks depending on what methods are used. NGS also does not depend on virus-specific primers and supports the “replacement, reduction, and refinement” recommendations regarding animal testing (24, 25).

NGS approaches have great potential to replace or complement traditional methods for virus identification in biotherapeutic and vaccine production. However, there are many possible options for the experimental and computational methods that can be used in an NGS experiment. To converge toward consensus approaches, these variations should be thoroughly tested and compared against each other and to the traditional virus detection procedures. Here, we investigate several different approaches for performing the bioinformatics analysis that is required to assign taxonomic information to sequencing reads. In brief, this analysis often involves finding homology between the sequencing reads from a sample and known viral sequences. There are several important decisions to consider before starting this analysis, including which viral reference database to use and whether or not to filter out host reads. There has been previous work in building databases that cover the diversity of viral sequences (different virus types, transposons, retroviral sequences, etc.), do not contain host sequences, and maintain a reasonable size for querying (26). In addition, host filtering is often recommended because it decreases the background noise caused by host genome reads when identifying virus sequences and speeds up the following classification of nonhost reads (27, 28).

Another key decision is whether to use full sequence alignment methods to find homology or to use *k*-mer-based approaches. Tools that rely on the full alignment of reads to a database of sequences include tools which use BLAST (29) or Bowtie (30) for alignment such as IMSA (31), PathoScope (32), VirusSeeker (33), and VirFind (34). In contrast, *k*-mer-based methods, including Kraken (35), Kraken2 (36), KrakenUniq (37), Clark (38), and Centrifuge (39), find exact matches between small substrings (*k*-mers) from the sequencing reads and sequences in a viral reference database. These short, exact matches can be computationally identified much faster than full read length nonexact alignments. As expected, *k*-mer-based approaches are often less sensitive and specific when identifying species in diverse metagenomics samples but are significantly faster than the full sequence alignment methods (28). The speed increase of these tools will shorten the overall time needed to detect a contaminant in the biotherapeutic production process, increasing the potential application of these methods as in-process tests performed throughout process development. Kraken-derived tools (Kraken, Kraken2, and KrakenUniq) and Centrifuge (using custom databases) have shown high precision and recall metrics in previous benchmarking studies (40, 41). Although these studies have compared the performance of *k*-mer classifiers on metagenomics samples containing tens to hundreds of bacterial species, the authors are unaware of any comparison among these tools for adventitious virus detection. Tool performance could vary between detecting viruses versus bacteria due to length and compositional differences between their genomes. For instance, virus genomes can be significantly shorter than bacterial genomes and often have slightly higher gene densities (42, 43), impacting the *k*-mer signatures created and used during read classification.

In addition, these metagenomic tools are traditionally used for the discovery and classification of microbes in environmental and medical samples. Samples from the biotherapeutic or vaccine production processes will contain many fewer unique species than these environmental or medical metagenomics samples, which can contain up to thousands of microbial species (44). Therefore, it is possible that these faster NGS approaches could reach suitable levels of sensitivity and specificity for this application, although detecting low-level contaminants, novel viruses, and viruses distantly related to known viruses could remain a challenge. Any virus that goes undetected during the production of biologics poses a potential risk, and the sensitivity of these methods thus requires further investigation using samples with lower levels of virus contamination than the ones used in this study.

Here, we test several existing metagenomics tools to see how well they perform on NGS data sets from viral spike-in studies (simulated and real) and to assess their time and memory requirements. Samples from these viral spike-in studies imitate samples that could be taken during a contamination event in the biotherapeutic production pipeline and contain up to five virus species. First, several tools were tested on simulated NGS data sets to evaluate their performance and speed. A reduced number of tools with the addition of host filtering were then applied to NGS data from viral spike-in studies done with HeLa cells (45), enabling comparison to other bioinformatics analysis methods. We also applied the tools to spike-in studies done in CHO-K1 cells (46), which allowed us to examine the impact of using different host reference genomes (Chinese hamster versus CHO-K1) for read filtering.

## RESULTS

### NGS simulation of viral spike-in data.

**FIG 1:**
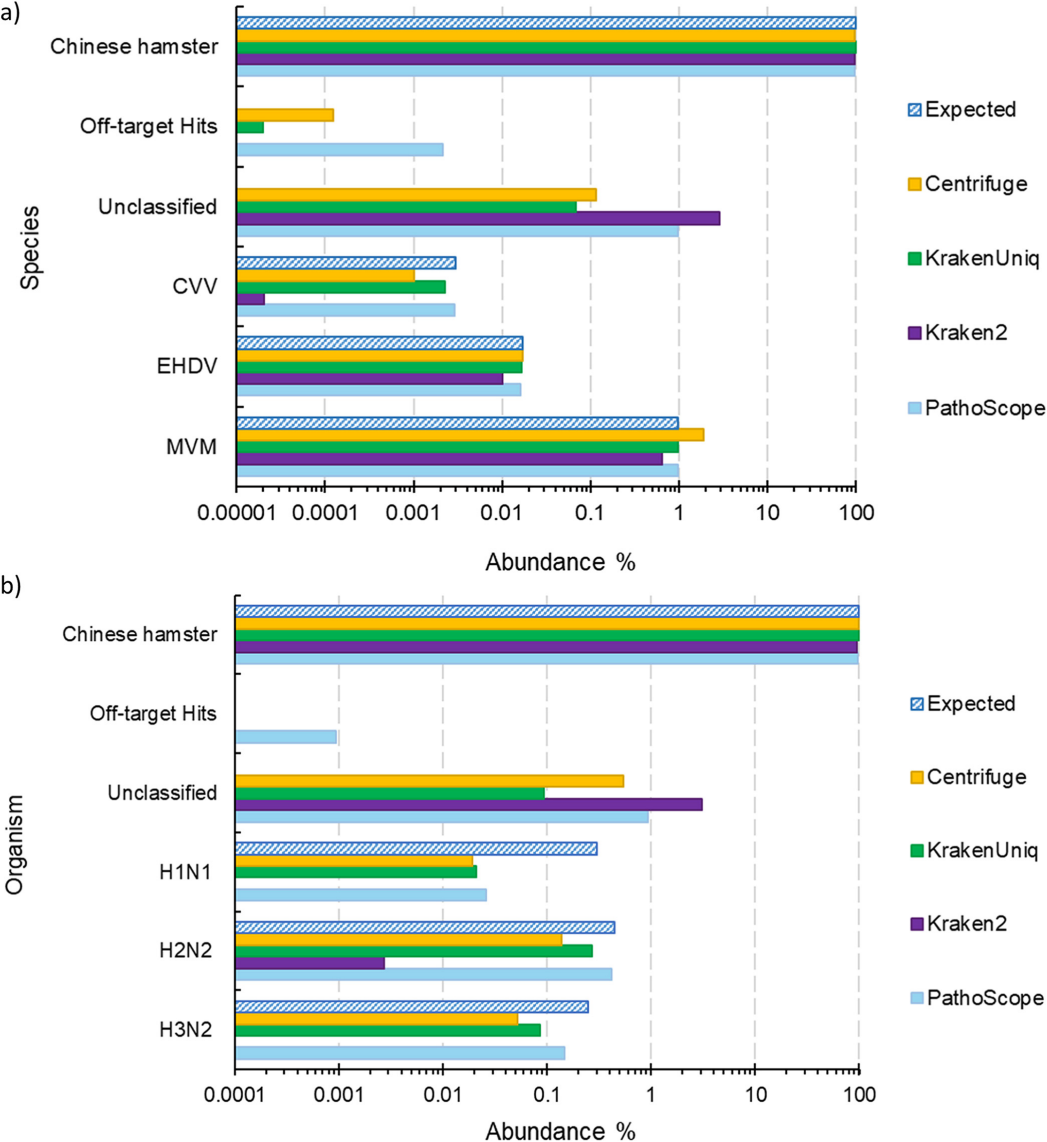
(A) Estimated abundances from various virus classification tools for the simulation 2 (a) and simulation 6 (b) data sets. The “Expected” category reflects the simulated abundance of each virus. The “Off-target Hits” category are reads that mapped to species that were not simulated.

Four tools were assessed on simulated NGS data, where various amounts of viral reads were simulated within a Chinese hamster (CH) host cell background. Read counts and percent abundances were visualized for each simulation data set for comparison across tools. We defined abundance as the number of reads classified for a species divided by the total reads after quality control. The results across simulation sets were generally consistent with all tools classifying the majority of reads correctly (Fig. 1a; see also Fig. S1 in the supplemental material). For simulation 1, which can be considered a negative control, Kraken2 and KrakenUniq had no reads mapping to off-target sequences, i.e., sequences from species that were not simulated, in the Reference Viral Database (RVDB), while Centrifuge only had one off-target read and PathoScope had 54 off-target reads. For the other simulations, PathoScope often incorrectly identified the most off-target species, while Kraken2 generally had the most reads without a taxonomic assignment (“unclassified”).

10.1128/mSphere.01336-20.6 FIG S1 Estimated species abundances from various virus classification tools for the simulation 1 no spike-in dataset (A), simulation 3 dataset (B), simulation 4 dataset (C), and simulation 5 dataset (D). The “Off-Target Hits” category are reads that could be classified but were not classified as Chinese hamster or one of the simulated viruses. Download FIG S1, TIF file, 0.7 MB. Copyright © 2021 MacDonald et al. 2021 MacDonald et al. https://creativecommons.org/licenses/by/4.0/ This content is distributed under the terms of the Creative Commons Attribution 4.0 International license.

We further evaluated the four tools by calculating the sensitivity, specificity, precision, accuracy, and the classification runtime of each tool (Table 1). KrakenUniq showed significantly higher sensitivity (0.93) than the other tools. Kraken2 showed perfect precision and specificity on the simulation data sets but had much lower sensitivity (0.13) than the other tools. KrakenUniq, Centrifuge, and PathoScope also maintained high specificity and precision (all >0.97). KrakenUniq, closely followed by Centrifuge and then PathoScope, had the highest average accuracy. The fastest tools were Kraken2 and KrakenUniq, followed by Centrifuge. The runtime of PathoScope was significantly higher than that of the other tools, taking 20 times longer than Centrifuge.

**TABLE 1:** Mean sensitivity, specificity, precision, accuracy, and runtime for each metagenomics tool across the simulated data sets^*a*^

Metric	Kraken2	KrakenUniq	Centrifuge	PathoScope
Sensitivity^*b*^	0.1339	0.9261	0.5924	0.4977
Specificity^*c*^	1.0000	0.9996	0.9977	0.9780
Precision^*d*^	1.0000	0.9999	0.9994	0.9979
Accuracy^*e*^	0.9704	0.9992	0.9986	0.9921
Time (h:min:s)^*f*^	00:00:25	00:01:45	00:05:59	01:45:55

a Each simulated data set consisted of approximately 10 million reads.

b Reads correctly classified as one of the expected viruses divided by the total reads classified at the species level.

c Reads correctly classified as not one of the expected viruses divided by the total reads classified as not one of the expected viruses.

d Reads that are correctly classified as one of the expected viruses divided by the total viral classifications made (unclassified reads are not included).

e Correctly classified reads divided by the total reads classified at the species level.

f Real time elapsed using 16 processors.

While we focused on how well the tools could identify viruses at the species level, we briefly investigated if they were able to distinguish between virus serotypes by running them on the simulation 6 data set. Simulation 6 consisted of reads from three different serotypes of influenza A, and PathoScope appears to be the best at distinguishing them (Fig. 1b), with the most reads classified at the strain level (59.64%). KrakenUniq classified 37.85% of the reads at strain level, whereas Centrifuge classified 20.90%.

Based on these results, KrakenUniq and Centrifuge were selected for further study on existing NGS data sets from viral spike-in studies performed in HeLa cells and CHO cells. Kraken2 was excluded from further tests due to its low sensitivity on the simulation data sets, and PathoScope was excluded due to its significantly longer computational time requirement than the other tools. KrakenUniq and Centrifuge also demonstrated reproducibility, producing consistent results when run on the same NGS data set multiple times (Centrifuge seed parameter set to the same value for each run).

### Analysis of HeLa cell spike-in study data.

Read counts and estimated percent abundances from KrakenUniq, Centrifuge, KrakenUniq after host filtering (KrakenUniq-HF), and BLASTn after host filtering (BLAST-HF) were compared to the results reported by Khan et al. (45). In that study, three different labs were requested to do their own sample preparation, sequencing, and bioinformatics analysis on virus spiked HeLa cells. This enabled a comparison to three different bioinformatics approaches.

### (i) KrakenUniq-HF is the most specific, while BLAST-HF is the most sensitive approach.

**FIG 2:**
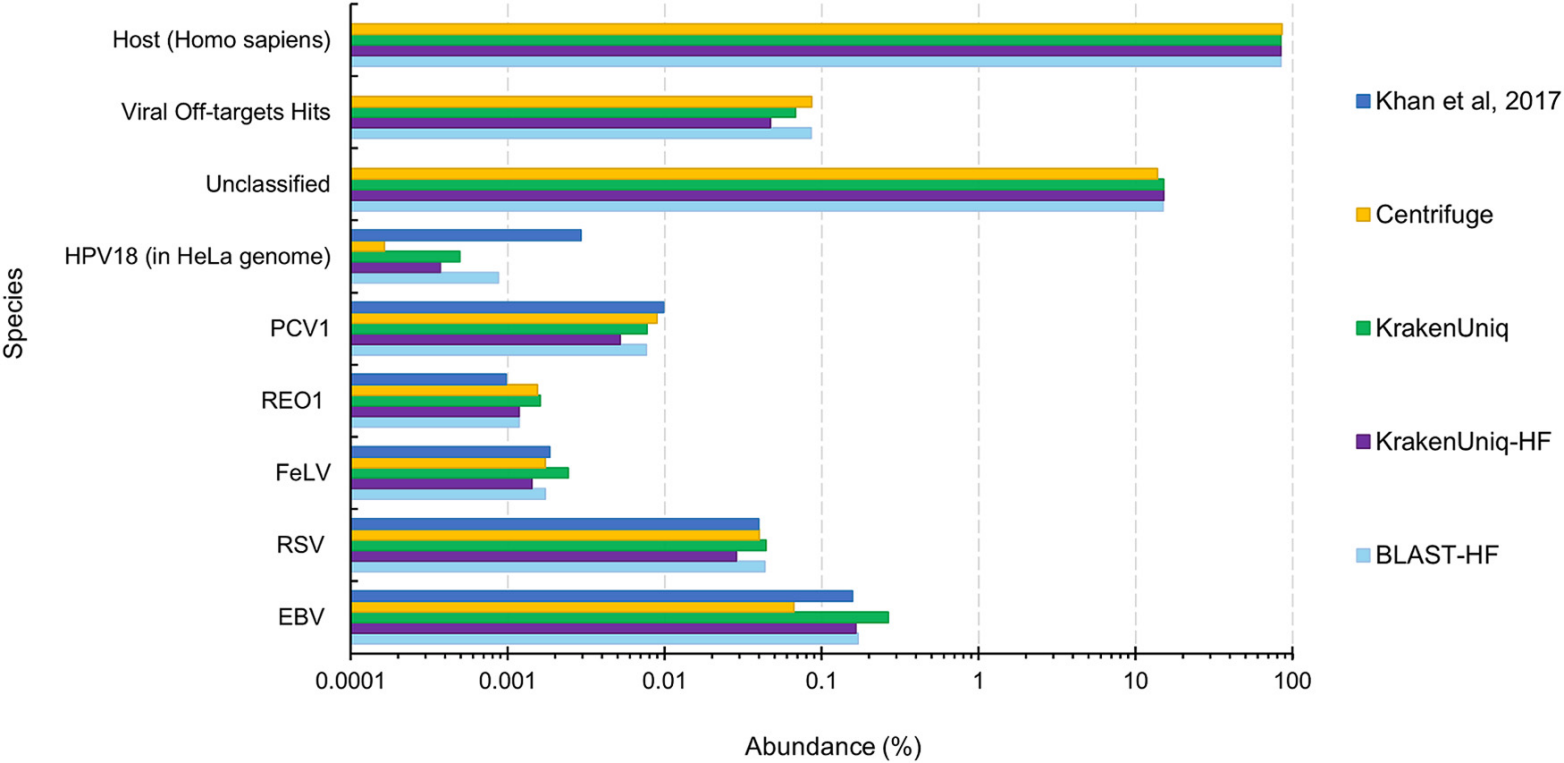
Estimated species abundances from various virus classification tools for the Lab A mixed sample (HeLa cell lysate with virus spiked in at 1 genome copy per cell) from Khan et al. (45). “HF” in the legend signifies that host filtering with KrakenUniq was done before classification against U-RVDB16. The “Unclassified” category refers to reads that could not be mapped to the host genome or U-RVDB16. The “Viral Off-targets Hits” category are reads that mapped to viruses other than the spiked-in viruses and human endogenous viruses. The “Host (Homo sapiens)” category for Centrifuge and KrakenUniq are reads that mapped to the human genome that was used in the reference database along with U-RVDB16. For KrakenUniq-HF and BLAST-HF, the “Host (Homo sapiens)” category are reads that mapped to the human genome during the filtering step.

The first lab, Lab A, used BLASTn to query the raw reads against a viral database from BioReliance/Millipore Sigma and confirmed viral hits against the NCBI nr/nt database. The estimated abundances from our methods for Lab A’s mixed spike-in sample, where virus was spiked-in at one genome copy per cell, show that the metagenomics tools work almost as well as using BLASTn on the full set of reads, even on relatively small sequencing data sets (Fig. 2). Most comparable were our results from using BLASTn to align the reads remaining after host (human) filtering to U-RVDB16, followed by Centrifuge and KrakenUniq without host filtering. All tools, though, were able to identify the expected viruses in approximately 2 million sequences, which is equivalent to the number of sequences produced from a typical MiSeq run. This suggests that biopharmaceutical companies may be able investigate a viral contaminant event using the less expensive and faster Illumina MiSeq platform, rather than the NextSeq or NovoSeq platforms, and detect viruses at the one or more genome copy per cell level. However, it is important to note that MiSeq would not have the depth of coverage to reach the limit of detection (LOD) needed for testing before cell bank or viral seed/product release.

For the no spike-in sample from Lab A, there were no off-target hits for either KrakenUniq or BLASTn with host filtering and a five-read cutoff (see Data Set S1, LabA-5). However, the rapid metagenomics methods for all spike-in samples resulted in off-target virus hits, i.e., they classified some of the reads as virus species that were not spiked-in (Table 2; see also Data Sets S1 to S3). KrakenUniq-HF was frequently the most specific with the fewest number of off-targets hits (Table 2; see also Data Sets S1 to S^3^), followed by BLAST-HF (see Data Sets S1 to S3). For each tool and sample combination, several hits were classified as nonviral, which is most likely due to misannotations in the reference database. There were also often viruses that were either closely related to the viruses expected or were human endogenous viruses (Table 2). Further investigation into the remaining off-target hits would be needed to determine whether they were due to misannotations in U-RVDB16, the mapping approach of the tool, or were actually present in the sample due to accidental contamination. Off-target read hits that are unique to a certain tool are most likely caused by the mapping approach of that tool. For Lab A mix, the majority of off-target viruses overlapped between tools suggesting that they are either real contaminants in the sample or due to misannotations in the RVDB. Examination of known misannotated sequences, i.e., sequences labeled with the incorrect species, in RVDB16 (https://rvdb.dbi.udel.edu/) suggested that several of the viral off-target hits for each tool were due to misannotations, such as the Semliki Forest virus, high island virus, white spot syndrome virus, and CRESS virus (Table 2). Another indication that these viruses were most likely identified due to misannotations was that they were identified across different samples from the three labs.

**TABLE 2:** Number of species identified by each classification tool for the Lab A mixed sample (HeLa cell lysate with virus spiked in at 1 genome copy per cell) from Khan et al. (45)

Category^*a*^	No. of species identified^*b*^			
	KrakenUniq	Centrifuge	BLAST†	KrakenUniq†
Total species	80	184	121	58
Total species*	34	41	51	30
Total viral species*	26	32	43	24
Viruses that are not closely related to those expected*	19	26	30	16
Viruses that are not closely related to those expected and are not human endogenous viruses*	19	26	29	16
Viruses that are not closely related to those expected, not human endogenous viruses, and not in an RVDBv16 misannotated sequence list*	9	15	17	9

a *, A five-read cutoff was used, meaning that a species required five or more read hits to be counted.

b †, After human filtering.

10.1128/mSphere.01336-20.1 DATA SET S1 Sample information and results of the different classification tools on Lab A’s spike-in samples from the Khan et al. study (45). Download Data Set S1, XLS file, 0.01 MB. Copyright © 2021 MacDonald et al. 2021 MacDonald et al. https://creativecommons.org/licenses/by/4.0/ This content is distributed under the terms of the Creative Commons Attribution 4.0 International license.

**FIG 3:**
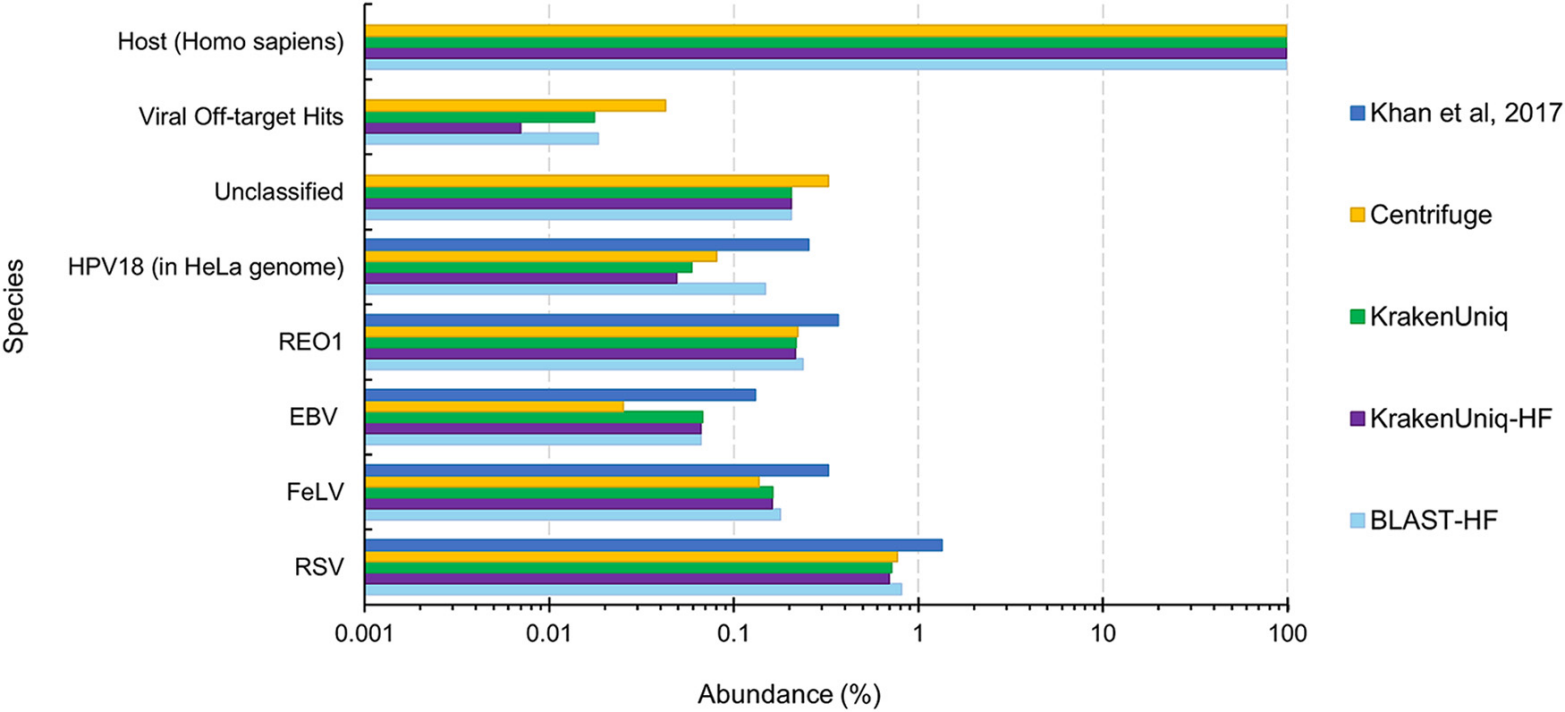
Estimated species abundances from various virus classification tools for LabC-1 sample (HeLa whole cells with viruses spiked in at 100 genome copies per cell) from Khan et al. (45). The “Unclassified” category refers to reads that could not be mapped to the host genome or RVDBv16.

**FIG 4:**
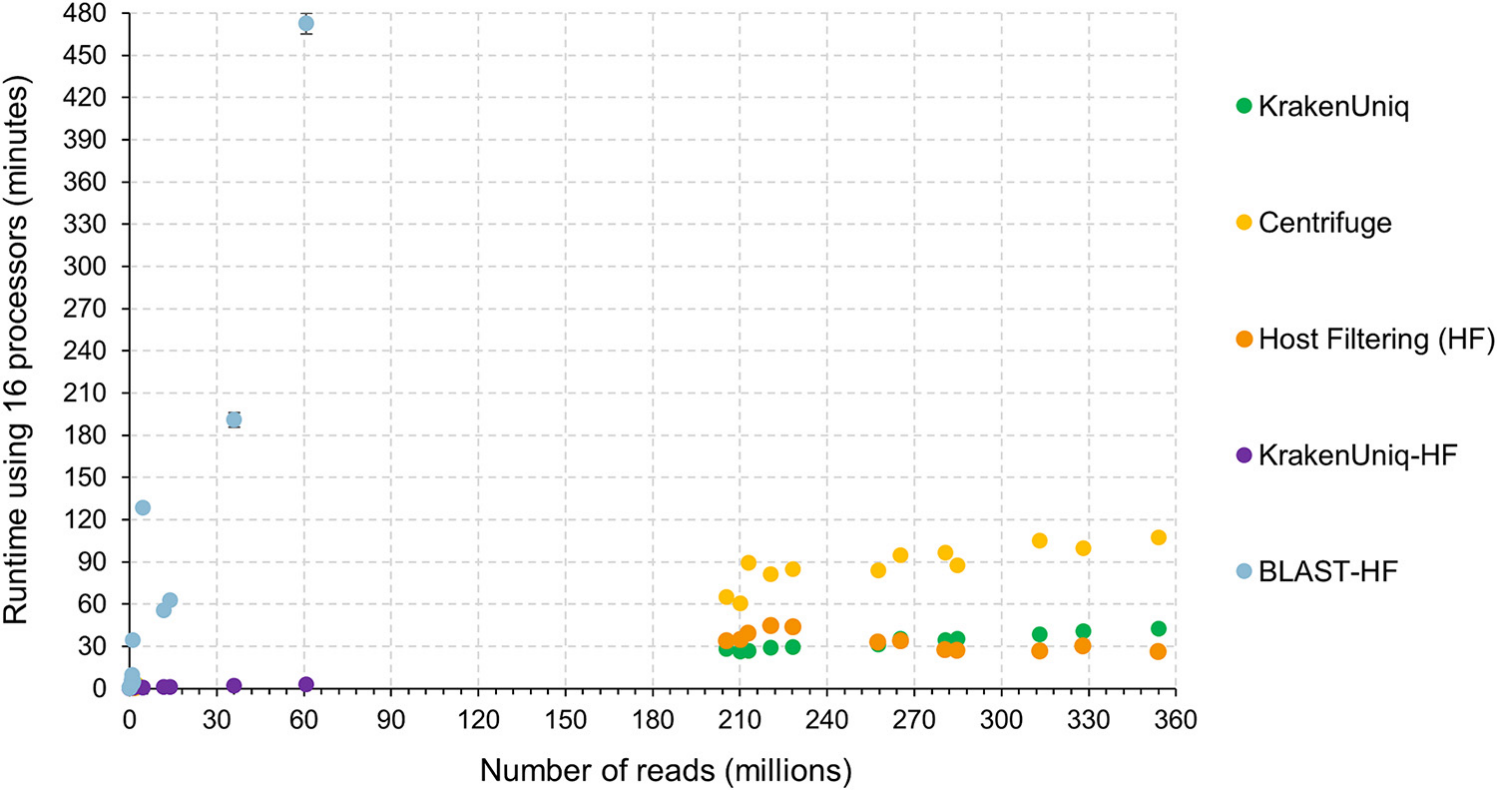
Runtimes for the tested tools when applied to samples from the HeLa cell viral spike-in study (45). Each tool was run three times on each sample using 16 threads on an AMD Opteron 6386 SE processor with 256 GB of RAM. KrakenUniq-HF and BLAST-HF are the times required by the KrakenUniq and BLAST, respectively, after host reads were filtered.

In addition to the results of Lab A, the results of the rapid viral detection tools on the other sequencing data sets showed the benefit of using BLASTn after host filtering (BLAST-HF) over using KrakenUniq after host filtering (KrakenUniq-HF). For instance, BLASTn abundance estimates and read counts were more similar to those from the bioinformatics analysis provided by Lab C, which involved mapping raw reads using BWA-MEM (47) for HPV18, Reo1, FeLV, and RSV (Fig. 3). However, the computational time requirement of BLASTn increases significantly as the number of reads increases (Fig. 4) and therefore using BLASTn for the viral identification portion is not always practical. BLASTn with fewer than 5 million sequences (reads or read pairs) required less than 20 min to run and therefore may be acceptable for a rapid turnaround application, but at higher sequencing depths the runtime of BLASTn could become prohibitive. If there are more than 5 million sequences after host filtering, KrakenUniq could be used instead. BLASTn and KrakenUniq both have linear increasing runtimes and thus linear regressions of the data shown in Fig. 4 can be used to extrapolate how long each tool will take for larger amounts of sequencing reads. Extrapolation to 1 billion reads predicts that KrakenUniq’s runtime will remain under an hour and half, while BLASTn will take 86 days. Although the number of parallel core jobs used to run BLASTn could be increased if the computer resources were available, for BLASTn to take the equivalent time KrakenUniq takes to analyze 100 million reads, BLASTn would need ∼64-fold more threads. This means that to get comparable times between running KrakenUniq with 16 threads and running BLASTn, BLASTn would need to be run using just over 1,000 parallelized core jobs.

KrakenUniq mapped slightly fewer reads (0.8 to 2.7%) to the expected viruses after host filtering (Fig. 3) compared to KrakenUniq without host filtering. One reason for this could be that there were host reads that were not filtered out and then erroneously mapped to one of the viruses. However, it is possible that some viral reads mapped to the host during host filtering and therefore were not available for mapping to the viruses. The latter, if true, could result in a slight loss of sensitivity for KrakenUniq-HF versus KrakenUniq for the identification of viruses at low abundances. For instance, at one genome copy per cell, the decrease in reads mapping between KrakenUniq and KrakenUniq-HF is 4.3 to 46.3% (see Data Set S3, LabC-3). This equates to a decrease of about 0.000008 to 0.00006 in percent abundance (8 to 60 reads per million), meaning that loss of detection of a virus would only happen if its percent abundance is less than this decrease which occurs at ∼0.86 genome copies per cell.

10.1128/mSphere.01336-20.3 DATA SET S3 Sample information and results of the different classification tools on Lab C’s spike-in samples from the Khan et al. study (45). Download Data Set S3, XLS file, 0.01 MB. Copyright © 2021 MacDonald et al. 2021 MacDonald et al. https://creativecommons.org/licenses/by/4.0/ This content is distributed under the terms of the Creative Commons Attribution 4.0 International license.

### (ii) Limit of detection of the rapid metagenomics approaches meet current industry standards.

**FIG 5:**
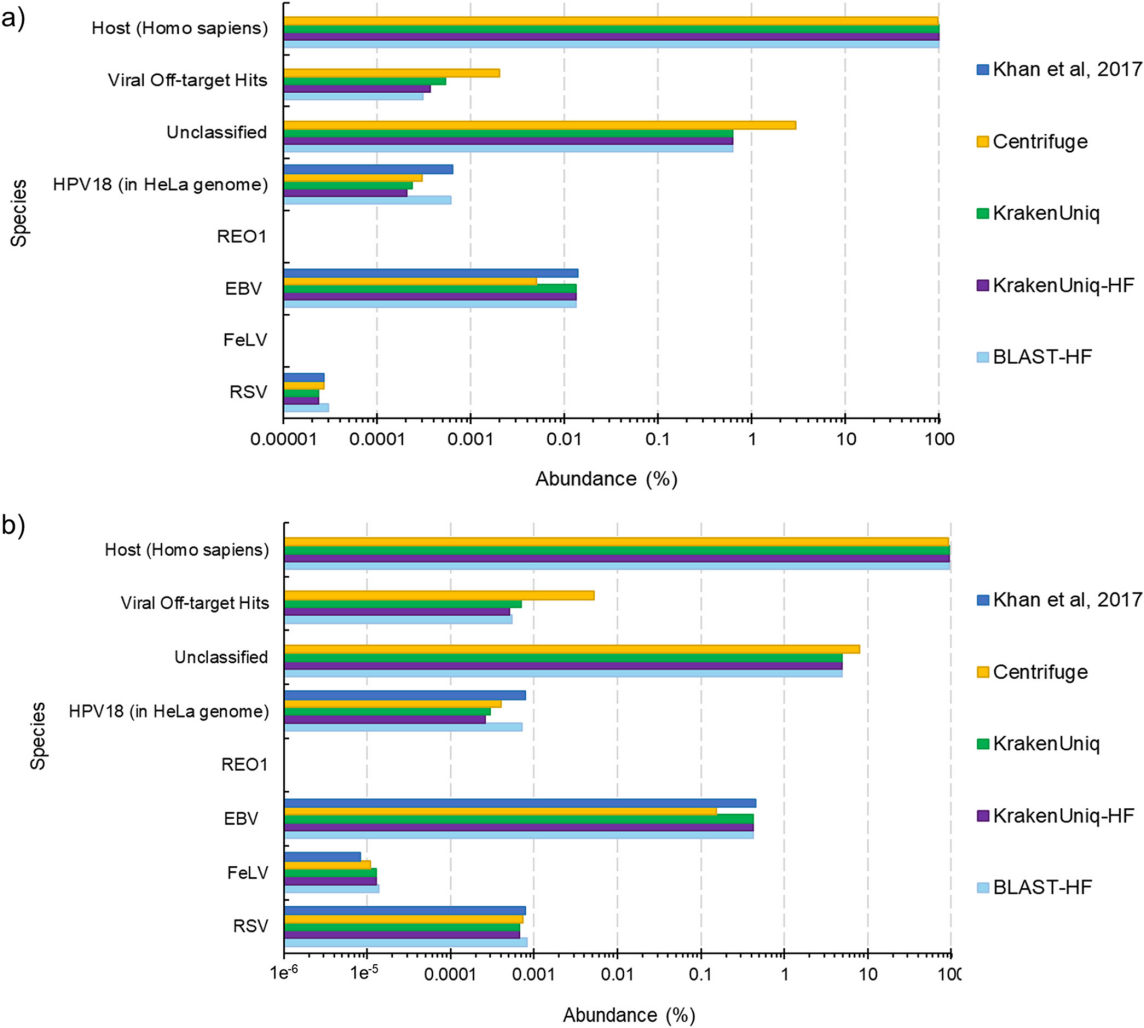
Estimated species abundances from various virus classification tools for the LabB-6 HeLa cell lysate sample with 0.1 genome copies of each virus per cell (a) and the LabB-5 HeLa cell lysate sample with 3 genome copies of each virus per cell (b). The “Unclassified” category refers to reads that could not be mapped to the host genome or RVDBv16.

**FIG 6:**
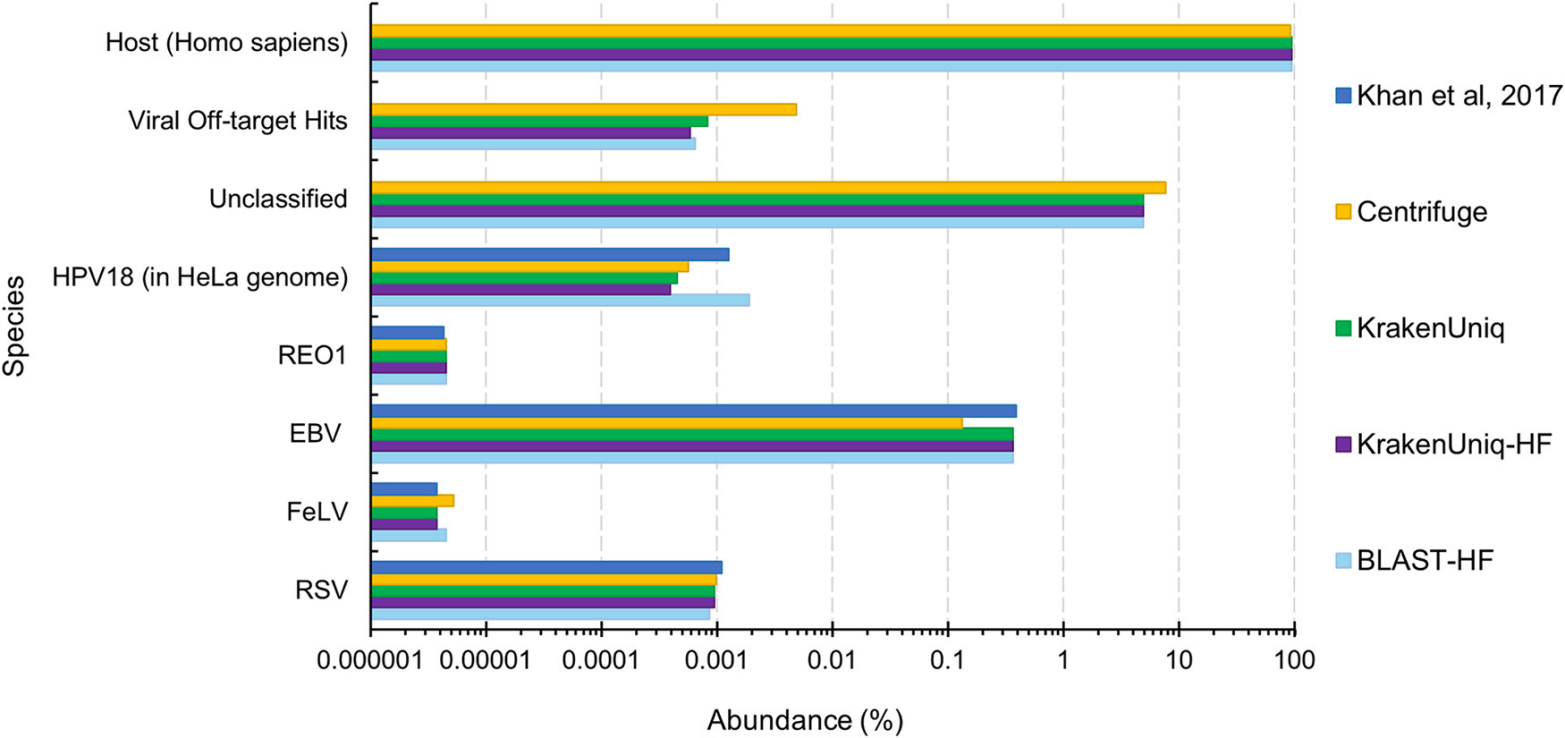
Estimated species abundances from various virus classification tools for the LabB-2 HeLa whole cell sample with three genome copies of each virus per cell. The “Unclassified” category refers to reads that could not be mapped to the host genome or RVDBv16.

Labs B and C in the Khan et al. study (45) systematically decreased the amount of viral spike-in in the samples to determine the LOD of their approaches. Therefore, we could compare the LOD of our bioinformatics analyses to each of these labs. For example, Lab B spiked in the four viruses in both HeLa whole cells (to simulate cell bank testing) and HeLa cell lysate (to simulate crude harvest) at 0.1, 3, and 100 genome copies per cell resulting in six different sequencing data sets. They used a bioinformatics approach that identified assembled contigs and remaining unassembled reads based on their phylogenetic distance from a set of reference sequences. The LOD for FeLV for our bioinformatics methods, as well as Lab B’s method, was between 0.1 and 3 genome copies per cell because no reads were identified as FeLV in the LabB-6 HeLa cell lysate sample which had viruses spiked-in at 0.1 genome copies per cell (Fig. 5a). The LODs of RSV and EBV for our approach and that of Lab B were <0.1 genome copies per cell as RSV and EBV reads were still identified in this sample. The LOD of Reo1 in HeLa lysate across all bioinformatics approaches, including that of Lab B, was between 3 and 100 genome copies per cells (Fig. 5b). For the HeLa whole cell samples, the results showed that the viruses had the same LODs as they did in the HeLa cell lysate samples, except that Reo1 was identified at three genome copies per cell (Fig. 6). These LODs are comparable to ones previously reported for detection of adventitious viruses using NGS (48) and fall within current industry standards for viral detection (13). The LODs are also similar to that of quantitative PCR (45, 49–51) in some cases, which is considered the gold standard technique for adventitious virus detection due to its low sensitivity (45). However, direct comparison among LODs of viral detection assays is difficult because LODs depend on the type of virus, the host background, and variations in the experimental protocol, including primer design, cycle parameters, reagents, and nucleic acid extraction methods for PCR-based assays (50, 52).

### (iii) Total viral read count correlates with spike-in concentration as expected.

**FIG 7:**
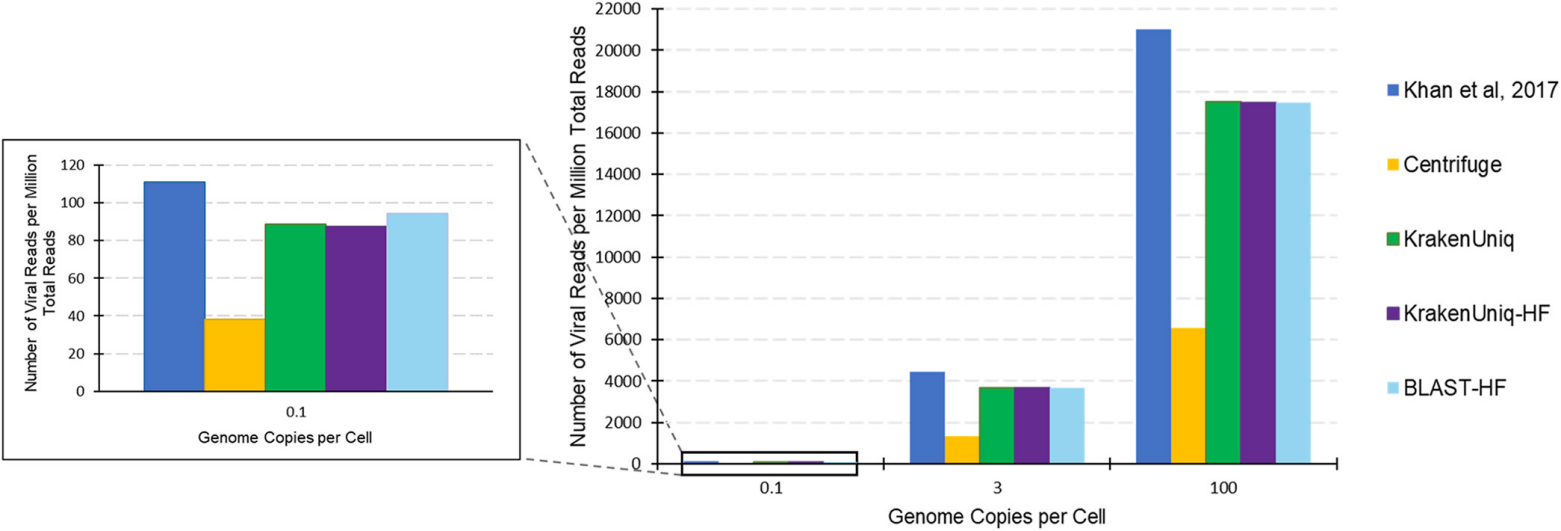
Viral read counts per million of the total sequencing reads for the HeLa whole cell samples from Lab B that received different amounts of viral spike-ins (Lab B samples 1 to 3). These spike-in samples consisted of RSV, FeLV, EBV, and Reo1, each spiked-in at the concentration on the *x* axis.

There is a clear downward trend in total estimated viral abundance as the amount of viral spike-in decreases as shown by Lab B whole cell spike-in samples 1 to 3 (Fig. 7). There is about a 45-fold difference in the number of reads classified as viral by the rapid metagenomics tools between the 0.1 viral genome per cell spike-in (∼20,000 reads) and the three viral genomes per cell spike-in (∼900,000 reads), and an ∼50-fold difference for Lab B’s bioinformatics approach. The number of reads classified as viral for the 100 viral genomes per cell spike-in (∼3,600,000 reads) was ∼4-fold higher than that of the three viral genomes per cell spike-in for both the approaches tested here and that of Lab B. Assuming the abundances from Lab B are the most accurate, BLASTn with filtering slightly out performs KrakenUniq without filtering, followed by KrakenUniq with filtering. Results from the tools on the other NGS data sets from Khan et al. (45) can be found in the supplemental material (see Data Sets S1 to S3 and Fig. S2 to S4).

10.1128/mSphere.01336-20.7 FIG S2 Estimated percent abundances from various virus classification tools for the LabA-5 (A), LabA-6 (B), LabA-7 (C), LabA-8 (D), LabA-9 (E), and LabA-10 (F) NGS datasets (45). “HF” in the legend signifies that host filtering with KrakenUniq was done before classification against U-RVDBv16. The “Unclassified” category refers to reads that could not be mapped to the host genome or U-RVDBv16. The “Viral Off-Target Hits” category are reads that mapped to viruses other than the spiked in virus and human endogenous viruses. Download FIG S2, TIF file, 1.2 MB. Copyright © 2021 MacDonald et al. 2021 MacDonald et al. https://creativecommons.org/licenses/by/4.0/ This content is distributed under the terms of the Creative Commons Attribution 4.0 International license.

### Analysis of CHO cell spike-in study data.

KrakenUniq, Centrifuge, KrakenUniq after host filtering, and BLASTn after host filtering were applied to NGS data sets from CHO cell viral spike-ins from Chiang et al. (46). This allowed us to gain insights on how well these tools work identifying viruses in samples with CHO cell host DNA, which is relevant for adventitious virus testing during the production of biotherapeutic proteins. We also investigated potential benefits of using both a CHO cell line-specific genome and the CH reference genome to filter out host reads.

**FIG 8:**
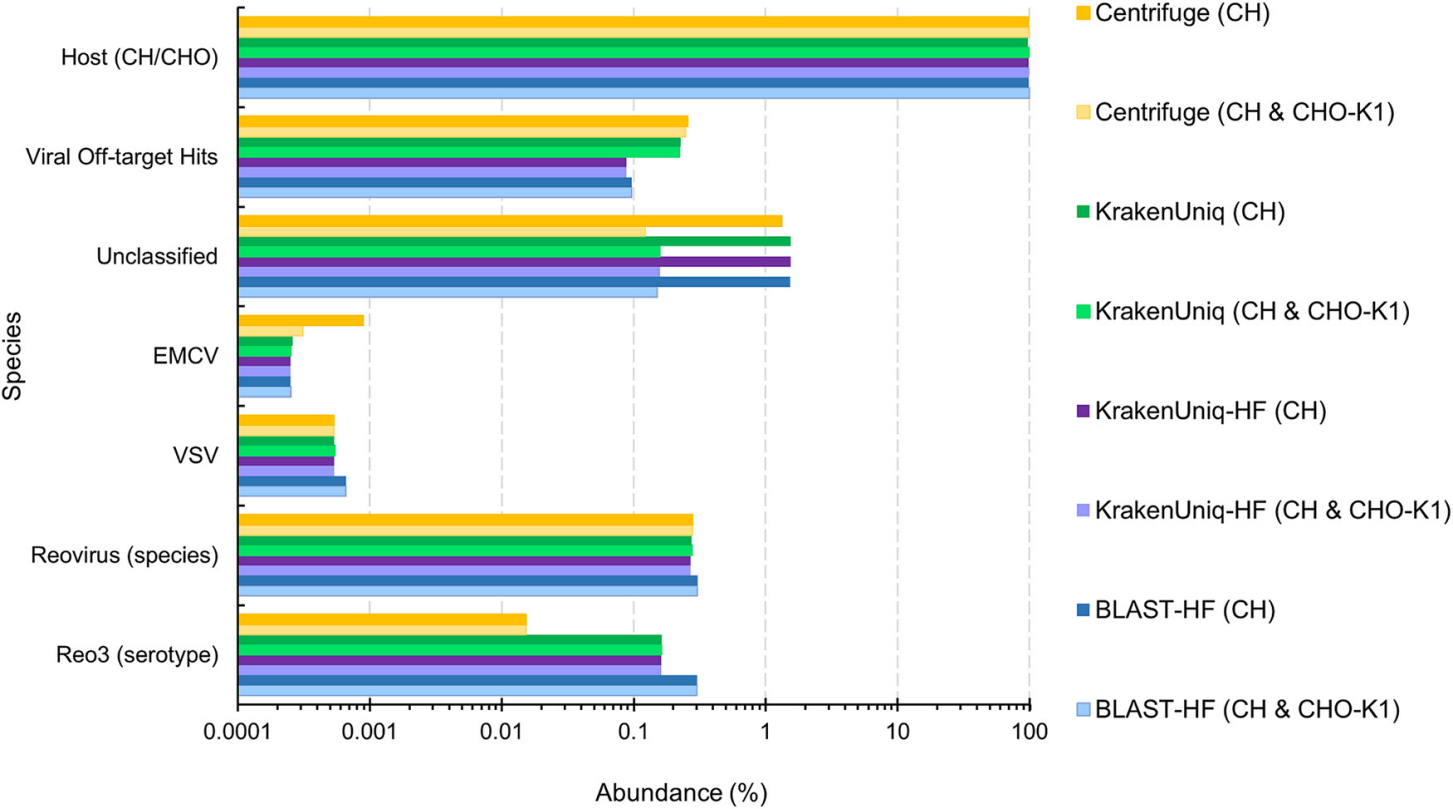
Estimated percent abundances from various virus classification tools for the reovirus-3 spike-in (experiment 1) NGS data set (46). The host reference genome was either the 2018 CH genome (CriGri-PICR, GCF_003668045.1) or both the 2018 CH and 2011 CHO-K1 (CriGri_1.0, GCF_000223135.1) genomes as distinguished in parentheses in the legend. “HF”’ in the legend signifies that host filtering with KrakenUniq was done before classification against U-RVDBv16. The “Viral Off-target Hits” category are reads mapping to viruses other than the spiked-in virus (reovirus) and the possible cross-contamination viruses (EMCV and VSV).

All tools were able to identify the viruses spiked into each sample (see Data Set S4 and Fig. S5). This includes reovirus, which was spiked-in at only 13 virus particles for every 10,000 CHO cells (Fig. 8). Interestingly, the tools also identified some potential cross-contamination of viral spike-ins. For instance, for the Reo3 spike-in (experiment 1), encephalomyocarditis virus (EMCV) and vesicular stomatitis virus (VSV) were also identified, albeit at low abundances (Fig. 8). In addition, while each tool estimated similar abundances of reovirus at the species level, they did vary on how well they identified the reovirus 3 serotype. KrakenUniq and BLASTn correctly identified the majority of reovirus reads as Reo3 (ca. 60 and 99%, respectively), while Centrifuge could only identify approximately 5.5% of the reovirus reads as the correct serotype (see Data Set S4).

10.1128/mSphere.01336-20.4 DATA SET S4 Sample information and results of the different classification tools on the spike-in samples from the Chiang et al. study (46). Download Data Set S4, XLS file, 0.01 MB. Copyright © 2021 MacDonald et al. 2021 MacDonald et al. https://creativecommons.org/licenses/by/4.0/ This content is distributed under the terms of the Creative Commons Attribution 4.0 International license.

10.1128/mSphere.01336-20.8 FIG S3 Estimated percent abundances from various virus classification tools for the LabB-1 (A), LabB-3 (B), and LabB-4 (C) NGS datasets (45). “HF” in the legend signifies that host filtering with KrakenUniq was done before classification against U-RVDBv16. The “Unclassified” category refers to reads that could not be mapped to the host genome or U-RVDBv16. The “Viral Off-target Hits” category are reads that mapped to viruses other than the spiked in viruses and human endogenous viruses. Download FIG S3, TIF file, 1.1 MB. Copyright © 2021 MacDonald et al. 2021 MacDonald et al. https://creativecommons.org/licenses/by/4.0/ This content is distributed under the terms of the Creative Commons Attribution 4.0 International license.

10.1128/mSphere.01336-20.9 FIG S4 Estimated percent abundances from various virus classification tools for the LabC-2 (A), LabC-3 (B), LabC-4 (DNA only) (C), LabC-5 (DNA only) (D), and LabC-6 (DNA only) (E) NGS datasets (45). “HF” in the legend signifies that host filtering with KrakenUniq was done before classification against U-RVDBv16. The “Unclassified” category refers to reads that could not be mapped to the host genome or U-RVDBv16. The “Viral Off-target Hits” category are reads that mapped to viruses other than the spiked-in viruses and human endogenous viruses. Download FIG S4, TIF file, 1.2 MB. Copyright © 2021 MacDonald et al. 2021 MacDonald et al. https://creativecommons.org/licenses/by/4.0/ This content is distributed under the terms of the Creative Commons Attribution 4.0 International license.

10.1128/mSphere.01336-20.10 FIG S5 Estimated percent abundances from various virus classification tools for the reovirus-3 spike-in experiment 2 (A), VSV spike-in experiment 1 (B), VSV spike-in experiment 2 (C), EMCV spike-in experiment 1 (D), and EMCV spike-in experiment 2 (E) datasets (46). The host reference genome was either the 2018 CH genome (CriGri-PICR, GCF_003668045.1) or both the 2018 CH and 2011 CHO-K1 (CriGri_1.0, GCF_000223135.1) genomes, as distinguished in parentheses in the legend. “HF” in the legend signifies that host filtering with KrakenUniq was done before classification against U-RVDBv16. The “Viral Off-target Hits” category are reads that mapped to viruses other than the spiked-in virus and the possible cross-contamination viruses. Download FIG S5, TIF file, 1.2 MB. Copyright © 2021 MacDonald et al. 2021 MacDonald et al. https://creativecommons.org/licenses/by/4.0/ This content is distributed under the terms of the Creative Commons Attribution 4.0 International license.

The results for each sample also show the benefit of using the CHO-K1 genome in addition to the 2018 CH genome. These two genome assemblies share about 95% coverage with 99.3% identity (https://www.ncbi.nlm.nih.gov/genome/annotation_euk/Cricetulus_griseus/103/). For all methods, the number of unclassified reads decreased almost 10-fold when using both genomes rather than just the 2018 CH genome. When using both genomes to filter out host reads, there is also a corresponding increase in reads identified as CH/CHO as expected. While the differences in read counts/abundances when using both genomes did not usually impact the final species counts for the tools, it did for the Reo3 experiment 1 spike-in sample. KrakenUniq with filtering using both genomes had one less off-target species identified than KrakenUniq using only the 2018 CH PICR genome (Table 3), suggesting that using both genomes for host filtering may reduce false positives. We also observed only a small increase in execution time and maximum memory usage when filtering with both genomes (see Data Set S4) with an average real-time increase of about 40 s with 16 threads and an average memory increase of 1.08 gigabytes.

**TABLE 3:** Number of species identified by each classification tool using the RNA sequencing data (SRR7779195) generated from the Reo3 experiment 1 spike-in sample (46)^*a*^

Category^*b*^	No. of species^*c*^							
	KrakenUniq	Centrifuge	BLAST†	KrakenUniq†	KrakenUniq‡	Centrifuge‡	BLAST§	KrakenUniq§
Total species	66	158	59	41	59	114	59	38
Total species*	39	53	23	21	34	47	23	19
Total viral species*	35	48	20	18	30	41	20	17
Viruses not closely related to reovirus*	33	46	17	16	28	39	17	15

a The host reference genome is either just the 2018 CH genome or both the 2018 CH genome and the 2011 CHO-K1 genome (see footnotes). The viral reference database used for all tools is U-RVDBv16.

b *, A five-read cutoff was used, meaning that a species required five or more read hits to be counted.

c †, After CH filtering; ‡, with CHO-K1 added to the reference database; §, after CH and CHO-K1 filtering.

### Assessment of divergent match sensitivity.

To further examine the read mapping sensitivity of KrakenUniq and BLASTn, reads were simulated from sequences that varied from an initial viral genome to reflect biodiversity that could be seen within a virus species. These reads were then mapped to the initial viral genome, which was the only sequence in the reference database for the two tools. The trends in read mapping counts were similar for the three viruses tested (see Data Set S5). For each virus genome, KrakenUniq was able to map more than 97% of the reads from sequences that were 95% or more similar to the viral genome. KrakenUniq also performed well for reads from sequences with 93% similarity, correctly mapping more than 85% of the reads. BLASTn had a higher sensitivity than KrakenUniq and was able to map more than 99% of the reads from sequences with 95% or more similarity. For reads from sequences with 93% similarity, BLASTn could map more than 93% of the reads to the viral genome.

10.1128/mSphere.01336-20.5 DATA SET S5 BLAST and KrakenUniq results from assessing divergent match sensitivity. Download Data Set S5, XLS file, 0.04 MB. Copyright © 2021 MacDonald et al. 2021 MacDonald et al. https://creativecommons.org/licenses/by/4.0/ This content is distributed under the terms of the Creative Commons Attribution 4.0 International license.

The simulated reads from the three viruses were also mapped to U-RVDB16 using KrakenUniq and BLASTn to examine the tools’ sensitivity when off-target sequences exist in the reference viral database (Table 4; see also Data Set S5). Overall, BLASTn showed better sensitivity than KrakenUniq for read mapping against the U-RVDB16, but KrakenUniq was still able to classify more than 90% of reads, simulated from sequences with approximately 95% similarity, as the correct species for all three viruses. In addition, at the 95% similarity level, 99% or more of the reads were mapped to the correct viral family. Even at the 90% similarity level, all reads either mapped to the expected viral family or they could not be classified, meaning that no reads were mapped to viruses outside the expected viral family. One unexpected result was the percentage of reads that mapped to the HIV-1 species by KrakenUniq using simulated reads from the sequence with 100% similarity to the HIV-1 genome (see Data Set S5). This percentage of reads was 80% rather than the 91 to 99% from sequences with the other similarity levels. This was the case for several simulated read sets and each time about 20% of the reads were classified into the correct genus but could not be further classified at the species level. These results suggest that both KrakenUniq and BLASTn are sensitive enough to classify the large majority of reads as the correct virus species, even when reads are from virus sequences with only 95% similarity to the corresponding viral sequence in RVDB.

**TABLE 4:** Average percentages of reads mapped to U-RVDB16 at each taxonomic level for various amounts of mutations simulated in the original virus genomes of HIV-1, MVM, and reovirus 3 Dearing

% Similarity to virus genome	Family (%)	Genus (%)	Species (%)	Unclassified (%)
KrakenUniq				
100	100	95.82	90.18	0
98.06	99.98	95.98	95.06	0.017
97.15	99.71	96.25	95.17	0.29
96.15	99.6	95.60	94.1	0.40
95.07	99.36	95.28	93.98	0.64
93.08	93.08	87.49	84.77	6.92
90.01	73.03	68.60	65.35	26.97
BLAST				
100	100	100	99.87	0
98.06	99.98	99.98	99.15	0.02
97.15	99.84	99.84	99.63	0.16
96.15	99.76	99.76	99.15	0.24
95.07	99.70	99.70	99.55	0.30
93.08	96.70	96.70	96	3.23
90.01	85.23	85.20	83.26	13.77

## DISCUSSION

The use of NGS for viral detection during the production of biotherapeutics and vaccines provides several benefits over existing viral detection methods and therefore, has potential to complement or replace the standard methods. Here, we investigated using metagenomics tools for the taxonomic classification of sequencing reads on simulated and public NGS data sets. If sequencing read classification can be completed quickly and accurately, the bioinformatics analysis portion of NGS could provide very rapid turnaround time for virus detection and in the future, could be set up as a web-based tool, providing a user-friendly, intuitive interface that does not require potential users to have command-line experience.

Several of the rapid *k*-mer-based approaches had high accuracy, sensitivity, and specificity metrics on the simulated data sets, which represented cases where the metagenomics samples contain only a few viruses in a host background. KrakenUniq and Centrifuge had the best performance metrics and were subsequently further tested on real NGS data sets from two previously published viral spike-in studies (45, 46). Results on the real NGS data sets suggest that the best approach to classify NGS reads quickly as viral/nonviral is to first filter host reads using KrakenUniq, followed by classification of the remaining reads using either KrakenUniq or BLASTn depending on the scale of remaining data. BLASTn showed slightly higher sensitivity than KrakenUniq. However, the time BLASTn takes to query NGS reads against the RVDB significantly increased based on the amount of NGS reads, becoming much greater than KrakenUniq’s runtime after approximately 5 million reads. Therefore, we suggest using a cutoff of 5 million reads to decide between using KrakenUniq or BLASTn for read classification after host filtering.

Ideally, a high-quality genome assembly for the specific cell line being used to produce biotherapeutics would be used for host read filtering. However, these high-quality cell line assemblies are often not available. Therefore, we investigated using the publicly available, high quality CH reference genome (53) alone and along with the lower quality CHO-K1 genome assembly (54) for host read filtering. Using both assemblies gave fewer unclassified reads, a slight increase in reads identified as host, and it decreased the amount of off-target viral hits in one of the samples examined in this study. As the quality of a given host cell’s genome assembly is improved (55), the performance of these approaches is expected to improve. There was also a minimal increase in time and memory when using an additional genome for host filtering. Thus, we suggest using a cell line-specific genome (such as one for CHO-K1, DG44, etc.) in addition to the high-quality CH reference genome if available. However, if the cell line genome is not available, using the CH genome alone to host filter is a viable option.

There are still several challenges facing the use of NGS for adventitious virus testing, including shortcomings that are specific to using rapid metagenomics methods to detect and classify viruses. One main challenge facing the rapid metagenomics approaches tested in our work will be to reduce the number of false-positive results without reducing sensitivity. Each positive result would need to be confirmed through additional assays, such as PCR, and thus false-positives could add to development timelines, which might be acceptable during upstream testing during early development but more difficult for testing of the final drug product. Thus, the false-positive rate also suggests that NGS approaches may be best suited for in-process viral testing rather than replacing current final release assays. Further updates to RVDB may help decrease incorrect classification of reads since the updates will involve the removal of nonviral sequences that may have previously been incorrectly classified as viral in the NCBI database or other sources. In addition, more analyses would help determine the most appropriate read cutoff, or other strategies, that could help reduce false positives. Overall, the selection of the cutoff value causes a trade-off between specificity and sensitivity with stringent cutoff values possibly filtering out viruses at low concentrations. A future method to investigate could be to apply a less-stringent cutoff to reads after they have been filtered based on mapping quality and/or position, enabling species with high quality and coverage to be highlighted. Another limitation of *k*-mer-based approaches is that they do not perform as well as full-read alignment methods regarding novel virus detection. If novel virus detection were of interest, using BLASTn as the virus classifier with low stringency parameters and in-depth hit follow up would be more suitable than using KrakenUniq as the classifier. However, PCR-based assays, which are currently used to detect adventitious viruses, have no ability to detect novel viruses.

Other challenges facing the use of NGS for viral detection include the fact that NGS detects the presence of viral nucleic acids and not actively replicating virus. For instance, NGS can detect remnant endogenous viral sequences that are often not cause for concern unless they can produce viral particles. In addition, if a viral contaminant has not been sequenced before or its sequence is not in the viral database being used in the pipeline, the virus could go undetected or be classified incorrectly. Nonetheless, there are many benefits to NGS for adventitious agent detection that make it an important approach for the industry.

Once a rapid metagenomics pipeline is set up, further investigation into the pipeline’s precise LODs for the virus types frequently seen in contamination events in various host cell backgrounds would be beneficial to provide users with an understanding of the pipeline’s capabilities and limits. For the viruses and protocols examined here, the LODs of the rapid metagenomics tools were all comparable to ones reported for NGS (21, 45, 48) and were comparable or better than LODs from other viral detection methods, including the most sensitive method currently used, qPCR (45, 49–51). However, it is important to note that the sensitivity of KrakenUniq and BLASTn for detecting small amounts of virus in a sample needs to be further investigated because all of the data sets examined in this study had relatively high quantity of virus, representing high levels of viral contamination.

Further inquiry into whether viral reads are being removed during host filtering would also be of interest. Reads from endogenous viruses would be filtered out during the process if they exist in the host genome sequence, but this can be prevented by masking the endogenous viral sequences. A remaining question is whether other types of viral reads map to similar regions in the host genome and would also be filtered out, obscuring detection of these viruses.

In conclusion, *k*-mer-based metagenomics approaches for read classification, specifically KrakenUniq, could be used as part of a fast pipeline to detect and classify known viruses in NGS data sets. These NGS data sets could be generated from samples taken at multiple points, such as cell banking and crude harvest, during the biotherapeutic production process. The specificity of these tools will continue to improve with the curation of the viral reference database and further investigation into read filtering parameters. Overall, fast, sensitive, and specific viral detection enabled by NGS analysis will facilitate safer and more efficient biomanufacturing of biologics.

## MATERIALS AND METHODS

### Reference database and taxonomic assignment.

Sequencing reads from each data set were assigned an NCBI viral taxonomic code at the species level by querying the unclustered Reference Viral Database version 16 (U-RVDB16) (26). U-RVDB16 contains approximately 2.8 million viral, virus-like, or retroviral sequences, excluding bacteriophages. The RVDB undergoes regular updates that are accessible from https://rvdb.dbi.udel.edu/, along with a list of misannotated sequences. Taxonomy codes were then converted into species names using the NCBI taxonomy dump files (downloaded on 15 October 2019), either by the tools themselves as described in their documentation (Kraken2, KrakenUniq, and Centrifuge) or by a custom script (PathoScope and BLASTn).

### NGS simulation of viral spike-in data.

After initial review of existing metagenomics tools, Kraken2, KrakenUniq, Centrifuge, and PathoScope were selected for testing on simulated NGS data sets. These tools were selected since they were able to use a custom reference database and appeared to be less computationally intensive than other available tools. Five million paired-end reads of 126 bp in length were simulated using InSilicoSeq v1.4.3 (56) with the provided Illumina HiSeq error model for six data sets. Reads were then trimmed using TrimGalore (www.bioinformatics.babraham.ac.uk/projects/trim_galore/) with a Phred quality score cutoff of 24 and a minimum length of 75 bp. For each simulation, the vast majority of reads (>99%) were created from the Chinese hamster (CH) reference genome (CriGri-PICR, GCF_003668045.1) (53) to simulate host background sequences. Various amounts of reads were created from reference viral genomes to simulate viral spike-ins (Table 5). Simulation 1 contained only reads from CH as a negative control. Simulations 2 to 4 contained reads simulated from the genomes of MVM, epizootic hemorrhagic disease virus (EHDV), and Cache Valley virus (CVV). These three viruses are known to infect CHO cells and have been found in previous contamination events. Simulation 5 contained reads from the genomes of EHDV and bluetongue virus, which are both from the *Reoviridae* family. Simulation 6 contained reads from three serotypes of influenza A: H3N2, H2N2, and H1N1. Each data set was run through PathoScope, Kraken2, KrakenUniq, and Centrifuge using default parameters and a reference database containing sequences from U-RVDB16 and the CH PICR genome. The mean sensitivity, specificity, precision, accuracy, and classification runtimes for each tool were calculated. For calculating average runtimes, each tool was applied to each data set three times and were run using 16 threads on machines with the same specifications (AMD Opteron 6386 SE processor, 16 cores, and 256 GB of physical memory) after reference database setup was completed. During the initial use of the software, overhead time is required for setup of KrakenUniq (several minutes) and PathoScope (several hours). These initial runs were not used in the calculation of runtimes.

**TABLE 5:** Numbers of reads and resulting percent abundances generated in each NGS simulation data set^*a*^

Test set	No. of reads (% abundance)								
	Total (after QC)	CH	MVM	EHDV	CCV	Bluetongue virus	H3N2	H2N2	H1N1
Sim1	9,984,072	9,984,072 (100)	0	0	0	0	0	0	0
Sim2	9,984,202	9,884,202 (99)	98,000 (0.98)	1,700 (0.017)	300 (0.003)	0	0	0	0
Sim3	9,984,202	9,884,202 (99)	98,000 (0.98)	1,100 (0.011)	900 (0.009)	0	0	0	0
Sim4	9,984,202	9,884,202 (99)	32,948 (0.33)	33,606 (0.34)	32,948 (0.33)	0	0	0	0
Sim5	9,984,042	9,884,202 (99)	0	41,933 (0.42)	0	57,907 (0.58)	0	0	0
Sim6	9,984,200	9,884,202 (99)	0	0	0	0	24,960 (0.25)	44,929 (0.45)	29,953 (0.30)

a The numbers of reads in simulation 2 (Sim 2) to Sim 5 for each virus were chosen to cover a range of viral abundances, while the numbers of reads in Sim 6 were selected to determine how well the tools could distinguish between viral strains at similar abundances.

### Analysis of HeLa cell spike-in study data.

The two tools that performed best on the simulation data, KrakenUniq and Centrifuge, were used to identify viruses in NGS data sets from virus spike-in studies described in Khan et al. (45). In these studies, three different labs performed various protocols to prepare and sequence virus spike-in samples, as well as unique bioinformatics methods to classify the resulting sequencing reads. In addition to identifying reads that could be classified as the virus(es) of interest, each lab identified reads that aligned to the human papillomavirus type 18 (HPV18) because it is integrated into the HeLa cell genome (57).

In brief, Lab A carried out single spike-ins of Epstein-Barr virus (EBV), human respiratory syncytial virus A (RSV), feline leukemia virus (FeLV), reovirus-1 (Reo1), and porcine circovirus 1 (PCV1) at an approximate ratio of one viral genome copy per cell (see Data Set S1, Lab A Sample Info). Lab A also spiked-in five viruses into a single sample. DNA and RNA in the samples were sequenced using a Roche 454 GS FLX, producing approximately 1.5 to 2 million single-end reads per sample. Lab A used BLASTn to align the raw reads to a proprietary virus database (BioReliance/Millipore Sigma) to classify the sequencing reads.

Lab B did two sets of low (0.1 genome copies per cell), medium (3 genome copies per cell), and high (100 genome copies per cell) mixed spike-ins of RSV, FeLV, EBV, and Reo1 (see Data Set S2, Lab B Sample Info). One set of spike-ins was done in HeLa whole cells, while the other set was done in HeLa cell lysate, resulting in six samples. They sequenced DNA and RNA from their samples using an Illumina HiSeq 1500, which resulted in 200 to 300 million paired-end reads per sample. For the bioinformatics analysis, Lab B first assembled the sequencing reads and then used the proprietary PhyloID software (58) to classify taxonomically the assembled reads, as well as any single reads that could not be assembled.

10.1128/mSphere.01336-20.2 DATA SET S2 Sample information and results of the different classification tools on Lab B’s spike-in samples from the Khan et al. study (45). Download Data Set S2, XLS file, 0.01 MB. Copyright © 2021 MacDonald et al. 2021 MacDonald et al. https://creativecommons.org/licenses/by/4.0/ This content is distributed under the terms of the Creative Commons Attribution 4.0 International license.

Lab C also did a low, medium, and high mixed spike-in of RSV, FeLV, EBV, and Reo1 similar to Lab B. However, they sequenced only DNA in one set of samples and only RNA in the other set (see Data Set S3, Lab C Sample Info). These samples were sequenced using an Illumina HiSeq 2500, which generated 250 to 400 million paired-end reads per sample. Lab C carried out targeted mapping with BWA-MEM (47) to align raw reads to the virus genomes of interest after filtering reads that aligned to the human reference genome. More information on the viral spike-ins and sequencing is described in Khan et al. (45).

For our analysis, reads in each NGS data set were trimmed by TrimGalore with a minimum quality value of 26 and minimum length of 65 bp. All data sets were run through KrakenUniq and Centrifuge using a reference database containing sequences from U-RVDB16 and the human reference genome, GRCh38. To test the benefits of host filtering, all data sets were also run through KrakenUniq using only the GRCh38 genome as the reference database. Reads that were not identified as human were then run through KrakenUniq or BLASTn using a reference database of U-RVDB16. Centrifuge was excluded from this analysis because it did not perform as well as KrakenUniq in classifying viral reads against the U-RVDB16 and the human reference genome. BLASTn was only used after human filtering and was not used to search against a reference database containing both U-RVDB16 and the human reference genome due to the intensive time requirement of BLAST. Runs for benchmarking the four methods were carried out three times for each sample on machines with the same specifications (AMD Opteron 6386 SE processor, 16 cores, and 256 GB of physical memory).

### Analysis of CHO cell spike-in study data.

Similar approaches to those explained above were applied to NGS data sets from Chiang et al. (46) (see Data Set S4, Sample Info). These data sets were derived from sequencing cell lysate samples from CHO-K1 cells that had been infected by one of three different RNA viruses: VSV, EMCV, or reovirus 3 (Reo3). The VSV spike-in resulted in a multiplicity of infection (MOI) of 0.003, meaning there were three virus particles for every 1,000 CHO cells. The EMCV and Reo3 spike-ins resulted in an MOI of 0.007 and 0.0013, respectively. More information on the viral spike-ins and RNA sequencing can be found in Chiang et al. (46).

For our analysis, reads from each data set were trimmed by TrimGalore with a minimum quality value of 26 and minimum length of 65 bp. All data sets were run through KrakenUniq and Centrifuge using a reference database containing sequences from U-RVDB16 and the 2018 CH PICR genome. In addition, all data sets were run through KrakenUniq and BLASTn after host read filtering. Host filtering consisted of using KrakenUniq with either a reference database containing only the 2018 CH PICR genome sequences or a reference database containing both CH PICR and CHO-K1 (GCF_000223135.1) (54) sequences. The remaining reads, which were not classified as CH reads, were then queried against U-RVDB16 using KrakenUniq or BLASTn. Again, Centrifuge was not used in this analysis because of its performance against the U-RVDB16 and the CH reference genome.

### Assessment of divergent match sensitivity.

The read mapping sensitivity of KrakenUniq and BLASTn was further investigated by simulating 100× coverage of reads from sequences created by generating mutations within a viral genome. The mutations were simulated using TreeToReads (59), producing sequences with various levels of percent similarity (90 to 100%) to the initial virus genome. The input genomes were Reo3 Dearing (GCA_006298385.1), human immunodeficiency virus 1 (GCF_000864765.1), and MVM (GCF_000838465.1). ART (60), within TreeToReads, was used to simulate 125-bp-long paired-end reads with the Illumina HiSeq2500 error profile for each mutated sequence. These reads were then mapped twice using KrakenUniq and BLASTn, once to a reference database containing only the initial virus genome and once to RVDBv16.
