# Supplementary figures and images for: k-mer-Based Metagenomics Tools Provide a Fast and Sensitive Approach for the Detection of Viral Contaminants in Biopharmaceutical and Vaccine Manufacturing Applications Using Next-Generation Sequencing
Source: mSphere. 2021 Apr 21;6(2):e01336-20. doi: 10.1128/mSphere.01336-20 (PMC8546726; doi:10.1128/mSphere.01336-20)

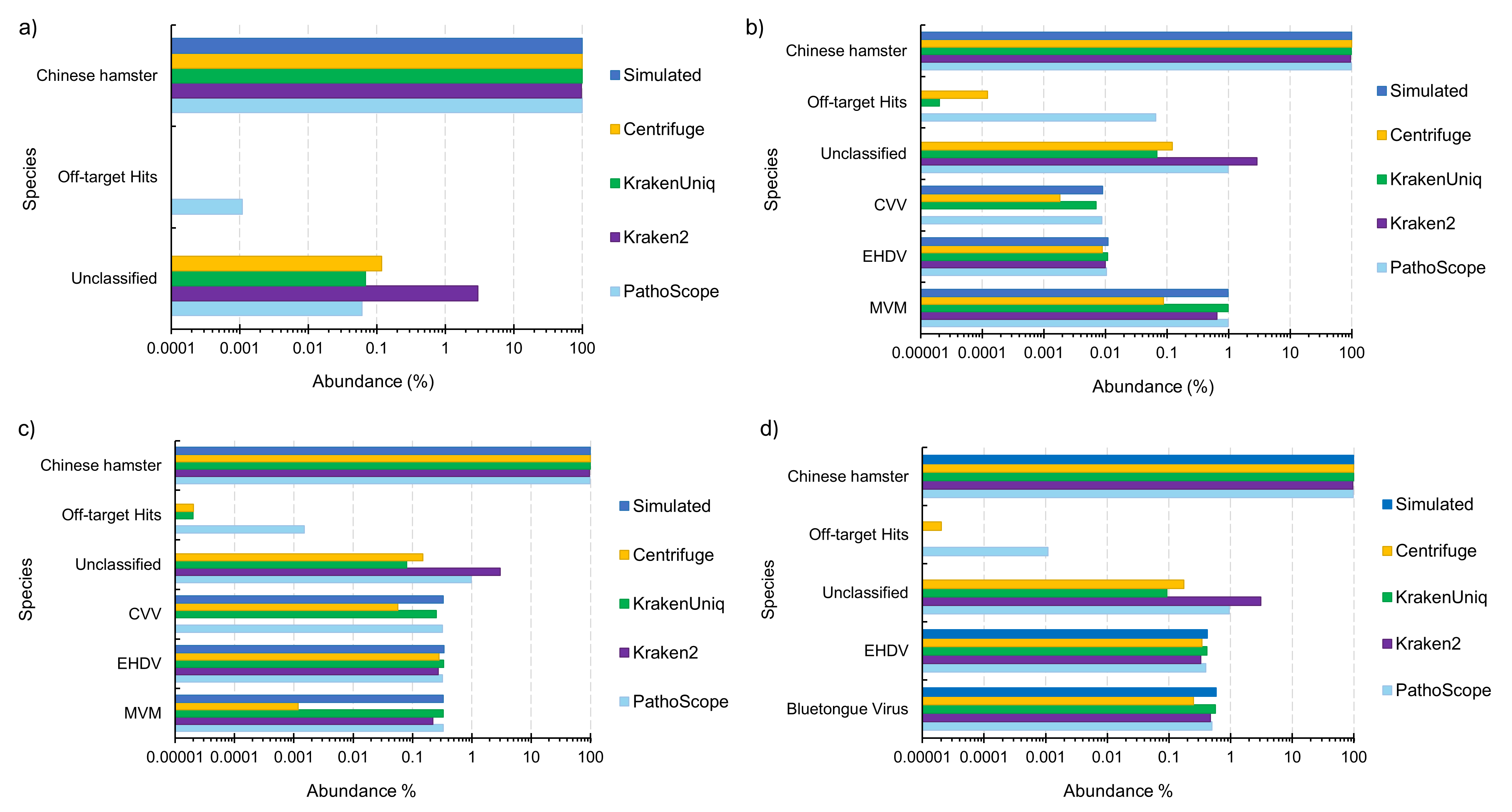

Supplement: FIG S1 [file msphere.01336-20-sf001.tif]

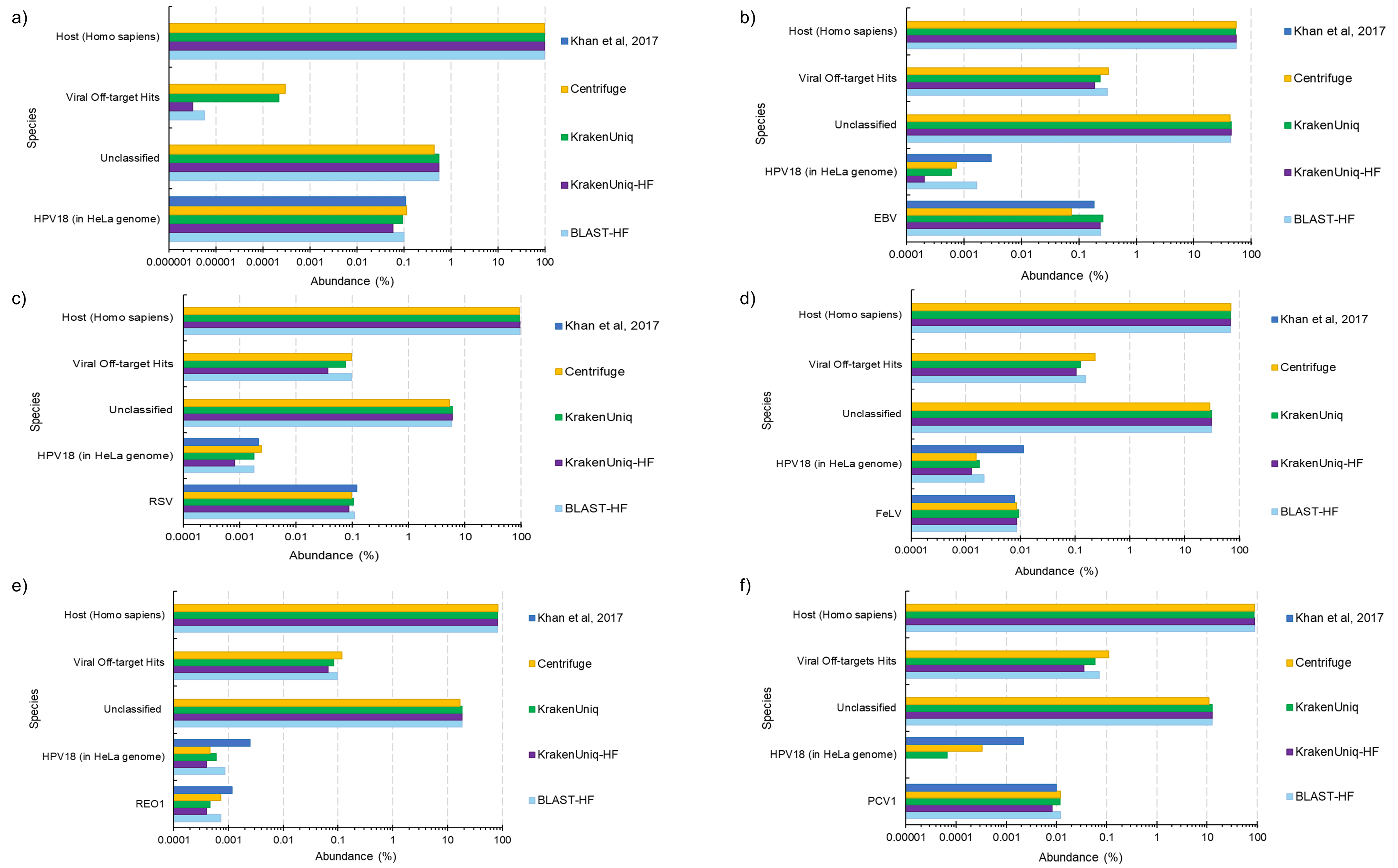

Supplement: FIG S2 [file msphere.01336-20-sf002.tif]

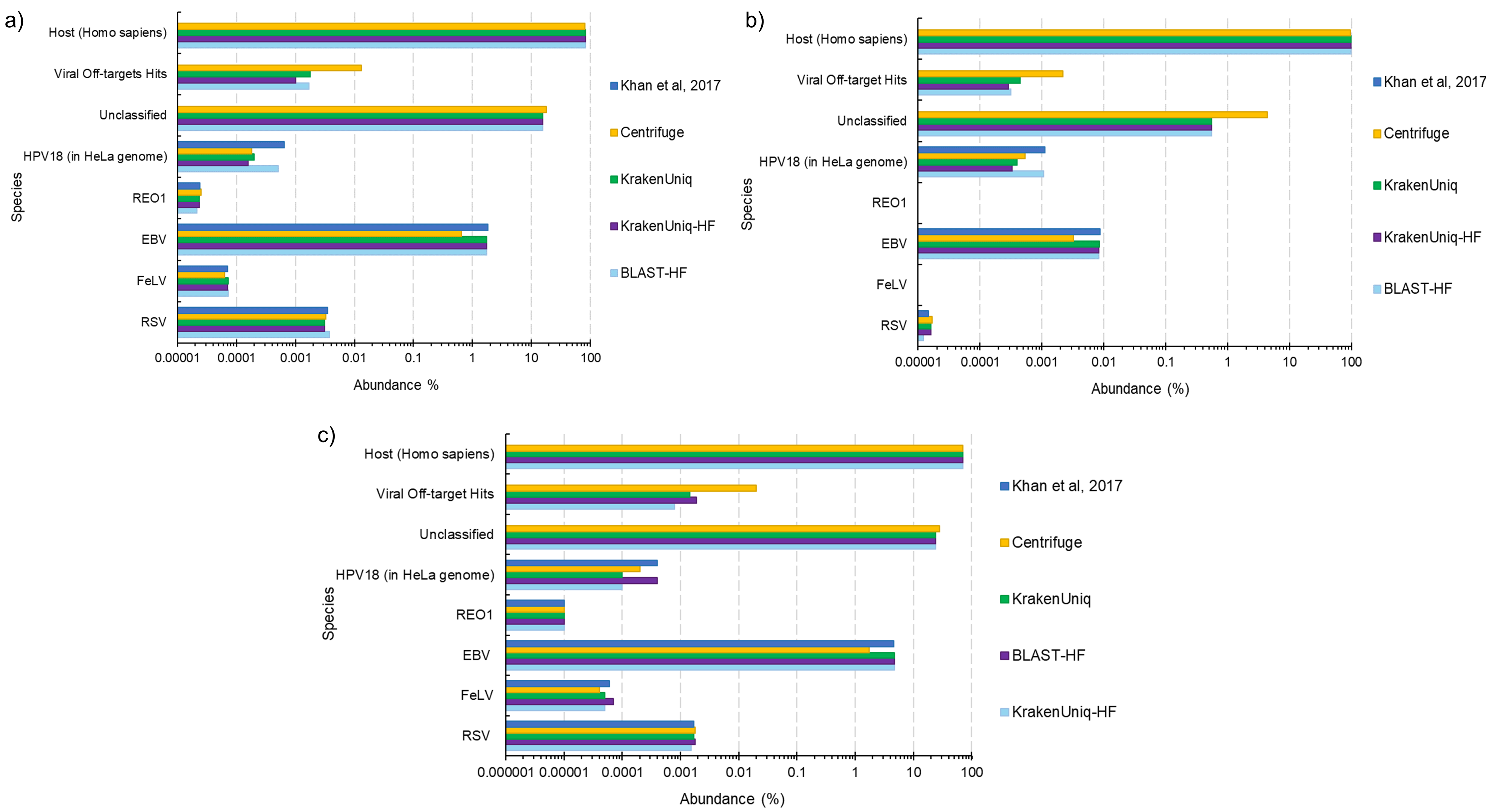

Supplement: FIG S3 [file msphere.01336-20-sf003.tif]

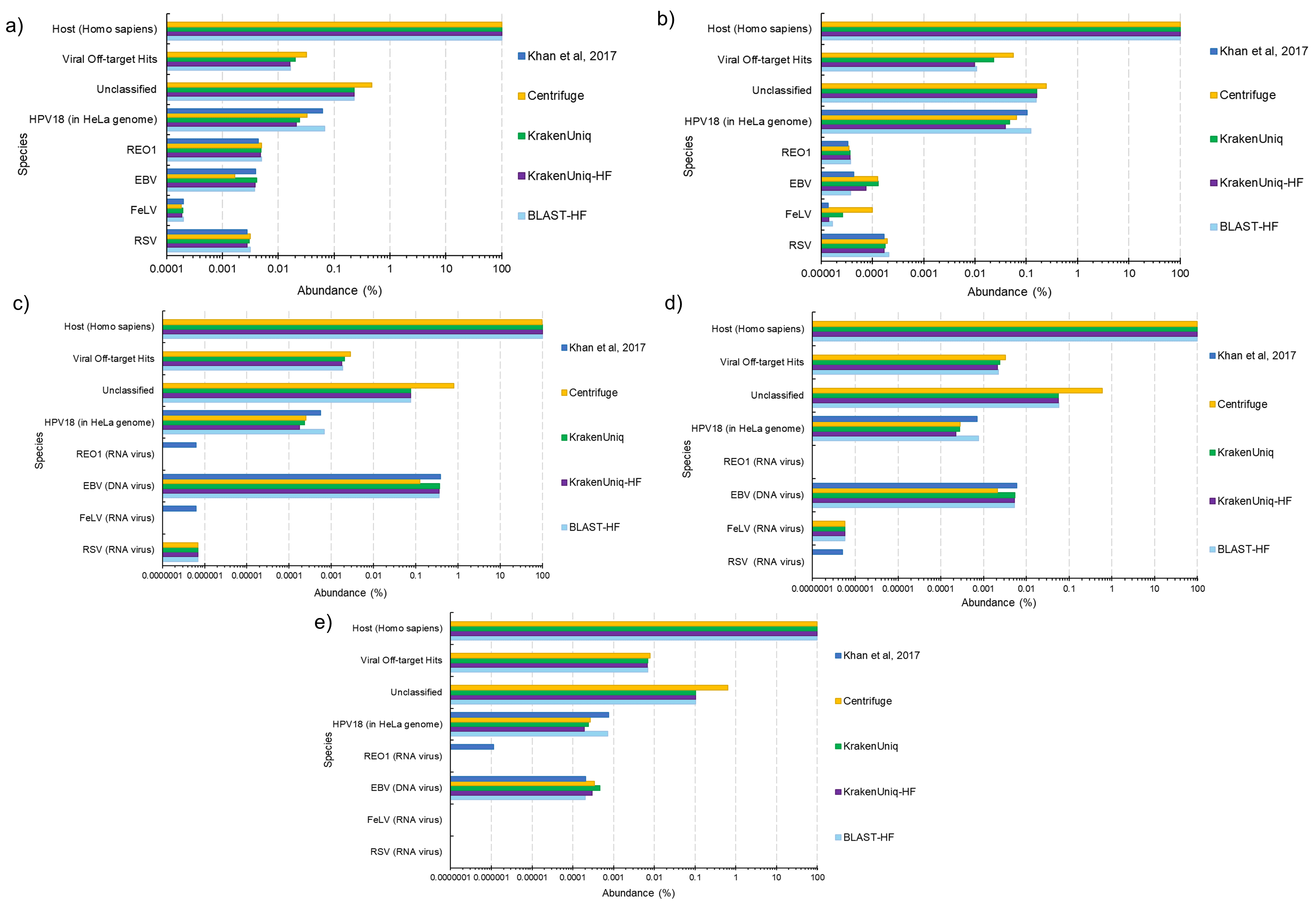

Supplement: FIG S4 [file msphere.01336-20-sf004.tif]

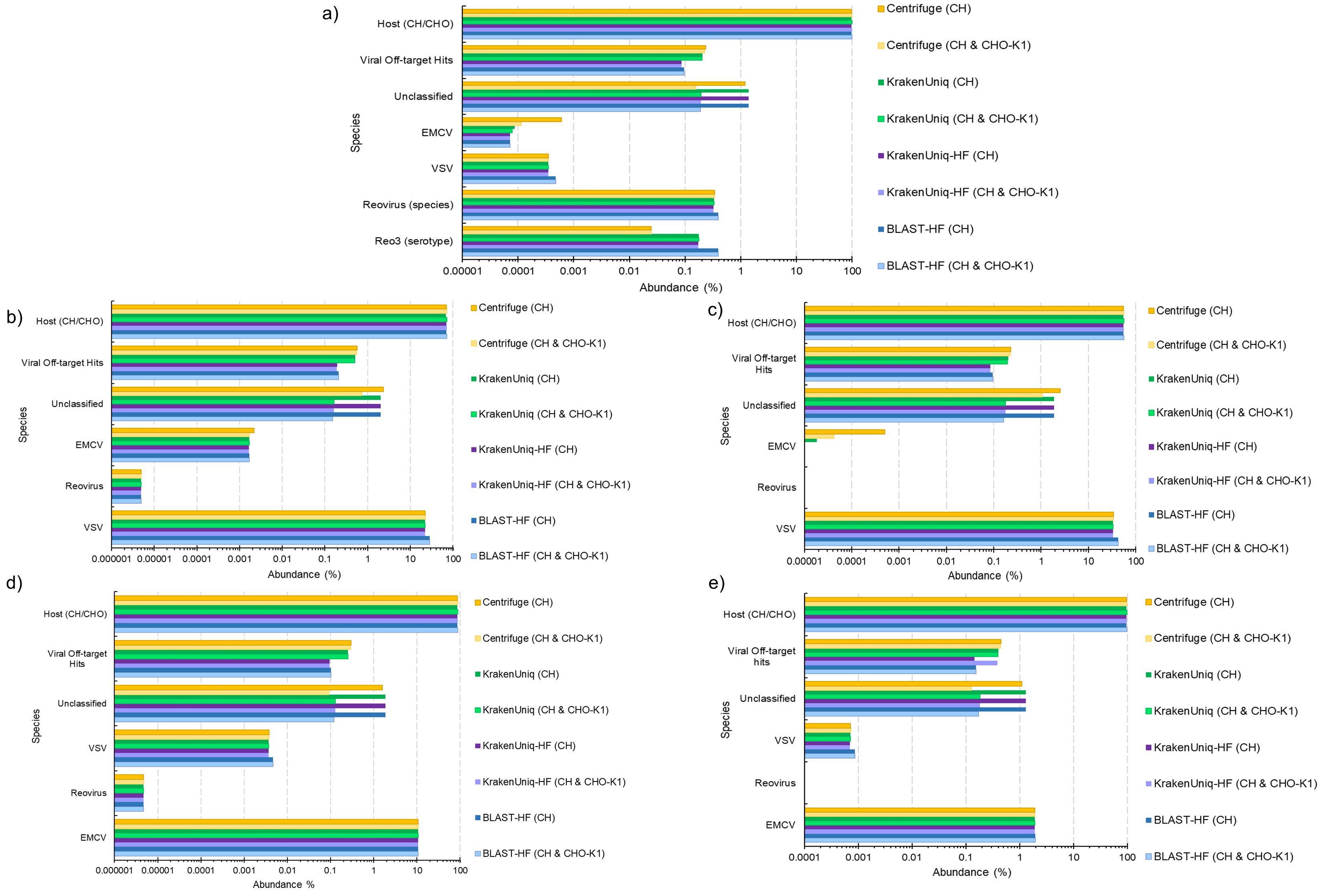

Supplement: FIG S5 [file msphere.01336-20-sf005.tif]
